# Melk facilitates pulmonary artery smooth muscle cell proliferation and migration in pulmonary hypertension via modulation of YAP/TAZ signaling

**DOI:** 10.3389/fcell.2025.1693346

**Published:** 2025-11-07

**Authors:** Qiang Yang, Peisheng Chen, Xiaoxia He, Jingting Jian, Miaomiao He, Tianxiao Lin, Shaoqiang Zheng, Degang Liu

**Affiliations:** 1 Department of Thoracic Surgery, The Third Affiliated Hospital of Southern Medical University, Southern Medical University, Guangzhou, China; 2 Department of Gastroenterology, The Third Affiliated Hospital of Southern Medical University, Southern Medical University, Guangzhou, China; 3 Department of Cardiovascular Surgery, Nanfang Hospital, Southern Medical University, Guangzhou, China; 4 Department of Respiratory Medicine, The Third Affiliated Hospital of Southern Medical University, Southern Medical University, Guangzhou, China

**Keywords:** pulmonary arterial hypertension, pulmonary artery smooth muscle cells, maternal embryonic leucine zipper kinase, MELK inhibitor, OTS167, cell proliferation, hippo-yap/taz signaling

## Abstract

Pulmonary arterial hypertension (PAH) is characterized by progressive pulmonary arteriolar constriction and remodeling, leading to elevated vascular resistance and right heart failure. Aberrant proliferation, migration, and phenotypic switching of pulmonary artery smooth muscle cells (PASMCs) are central to this process. Maternal embryonic leucine zipper kinase (MELK), a serine/threonine kinase of the AMPK family, is known to regulate cell cycle and tumorigenesis, but its role in PAH remains unclear. MELK expression was elevated in PASMCs from patients with PAH, in PDGF-BB–stimulated human pulmonary artery smooth muscle cells (HPASMCs), and in PASMCs of Su/H mouse lungs, indicating conserved upregulation across human and experimental models. *In vitro*, pharmacological inhibition or genetic silencing of MELK suppressed DNA synthesis, proliferation, and migration of HPASMCs under basal and PDGF-BB–stimulated conditions, concomitant with downregulation of PCNA and Cyclin D1. Conversely, MELK overexpression promoted PASMC growth and migration and accelerated the transition from a contractile to a synthetic phenotype. Mechanistically, MELK reduced YAP phosphorylation (Ser127), thereby activating Hippo–YAP/TAZ signaling and increasing downstream effectors (CYR61, CTGF, Birc5, Cyclin E), while leaving upstream gene transcription unchanged. The YAP inhibitor Verteporfin blunted MELK-driven PASMC proliferation and migration, underscoring the central role of YAP/TAZ signaling. Finally, *in vivo* pharmacological inhibition of MELK by OTS167 markedly reduced right ventricular systolic pressure, hypertrophy, and pulmonary vascular remodeling in Su/H mice, confirming the therapeutic relevance of MELK targeting in PAH. Collectively, these findings identify MELK as a novel regulator of PASMC pathobiology in PAH and suggest that it may represent a potential therapeutic target.

## 1 Introduction

Pulmonary arterial hypertension (PAH) is a progressive and life-threatening disorder characterized by sustained pulmonary arteriolar constriction and structural remodeling, which together lead to chronically elevated pulmonary vascular resistance (PVR), increased pulmonary arterial pressure, and ultimately right ventricular (RV) hypertrophy and failure (Lai et al., 2014; Thenappan et al., 2018). Although PAH is relatively uncommon, with an estimated prevalence of 15–50 cases per million (Humbert et al., 2022), it disproportionately affects young to middle-aged women and is associated with poor prognosis. Without therapy, the median survival after diagnosis is less than 3 years (Humbert et al., 2022).

A hallmark of PAH is the excessive proliferation and migration of pulmonary artery smooth muscle cells (PASMCs), which drive medial hypertrophy, neointimal formation, and the development of plexiform lesions (Southgate et al., 2020; Thompson and Lawrie, 2017). These maladaptive changes progressively narrow or obliterate small pulmonary arteries, impose an increased afterload on the RV, and accelerate functional decline. In addition, PASMCs undergo phenotypic switching from a contractile to a synthetic state, characterized by enhanced extracellular matrix deposition and vascular stiffening (Ma et al., 2023), which further aggravates pulmonary vascular remodeling. Targeting PASMC hyperplasia and phenotypic plasticity is therefore considered a key strategy to attenuate vascular remodeling in PAH.

Maternal embryonic leucine zipper kinase (MELK) is a serine/threonine kinase of the Snf1/AMPK family that regulates diverse cellular processes, including proliferation, apoptosis, stemness, and metabolism (Tang et al., 2020). MELK promotes tumorigenesis and progression via activation of PI3K/Akt/mTOR signaling, stabilization of EZH2, histone methylation, and regulation of the tumor immune microenvironment (Xu et al., 2020; Li et al., 2019; Yu et al., 2024; Tang et al., 2024). Overexpression of MELK enhances cell proliferation, migration, invasion, and therapy resistance, whereas pharmacological inhibition with agents such as OTS167 suppresses stem-like cell renewal, reverses drug resistance, and sensitizes cells to targeted therapies (Stefka et al., 2016; Eisfelder et al., 2021). Given its central role in driving abnormal cell proliferation and migration in cancer, MELK may have broader relevance in proliferative vascular diseases. However, its function in pulmonary arterial hypertension, particularly in pulmonary artery smooth muscle cell remodeling, has not been reported.

The Hippo–YAP/TAZ signaling pathway is a highly conserved regulator of organ size, tissue homeostasis, regeneration, and mechanotransduction (Moya and Halder, 2019). In its active state, the core kinases MST1/2 and LATS1/2 phosphorylate the transcriptional coactivators YAP and TAZ, leading to their cytoplasmic retention and proteasomal degradation, thereby preventing transcription of pro-proliferative and pro-survival genes (Kiang et al., 2024). Inactivation of the Hippo pathway results in YAP/TAZ dephosphorylation, nuclear translocation, and activation of downstream targets such as CYR61 and CTGF, which drive cell proliferation, migration, extracellular matrix remodeling, and resistance to apoptosis (Moya and Halder, 2019; Kiang et al., 2024). Aberrant Hippo–YAP/TAZ signaling has been implicated in various pathological processes, including cancer progression, tissue fibrosis, and aberrant vascular remodeling (Kiang et al., 2024). Recent studies have demonstrated that YAP/TAZ signaling is constitutively activated in PAVSMCs from patients and experimental models of PAH, promoting cell proliferation and survival, whereas YAP inhibition ameliorates pulmonary arterial remodeling (Kudryashova et al., 2016; Wang et al., 2019; Kudryashova et al., 2022). These findings highlight a critical role of Hippo–YAP/TAZ dysregulation in PAH pathogenesis and provide a mechanistic rationale for investigating its upstream regulatory factors.

We hypothesized that MELK promotes PASMC proliferation, migration, and phenotypic switching in PAH through activation of the Hippo–YAP/TAZ pathway. To address this, we employed a Sugen/hypoxia (Su/H) mouse model of PAH and conducted *in vitro* studies using human PASMCs to determine the effects of MELK inhibition with OTS167 and MELK overexpression on vascular remodeling, PASMC behavior, and Hippo–YAP/TAZ signaling. This study aims to elucidate the role and underlying mechanisms of MELK in PAH and to evaluate its potential as a therapeutic target for attenuating pulmonary vascular remodeling and disease progression.

## 2 Materials and methods

### 2.1 Animal studies

All animal protocols were approved by the Animal Research Policies of the Southern Medical University Committee in Nanfang Hospital (approval number NFYY-2022-0429) and complied with the Guide for the Care and Use of Laboratory Animals of the National Institutes of Health in China. Male C57BL/6J mice (8–10 weeks, 20–25 g; Cyagen Biosciences, Suzhou, China) were used. Mice were housed in a specific pathogen–free facility (22 °C ± 2 °C, 40%–60% humidity, 12 h light/dark cycle) with *ad libitum* access to standard chow and water and environmental enrichment. The Sugen 5416/hypoxia (Su/H) mouse model of pulmonary hypertension was established as previously described (Mouraret et al., 2013; Born et al., 2023). Mice received intraperitoneal injections of Sugen 5416 (20 mg/kg; MedChemExpress, dissolved in 0.5% carboxymethylcellulose containing 0.4% polysorbate 80) once weekly for 3 consecutive weeks and were housed in a normobaric hypoxia chamber (10% O_2_, balanced with N_2_) for a total of 4 weeks. Beginning at the end of week 1, mice were randomized (computer-generated sequence) to saline or the MELK inhibitor OTS167 (5 mg/kg, intraperitoneally, once daily) for 3 weeks. Investigators performing outcome assessments were blinded to group allocation.

### 2.2 Right ventricular systolic pressure (RVSP) measurement

Mice were anesthetized with inhaled isoflurane (induction at 3%–4%, maintenance at 1.5%–2% in oxygen) and placed on a temperature-controlled heating pad (37 °C). A 1.4F micro-tip pressure catheter (Millar Instruments) was inserted into the right jugular vein and advanced into the right ventricle under continuous pressure monitoring. The zero reference was set at the mid-thoracic level. Stable pressure waveforms were recorded for ≥30 s and RVSP was calculated as the mean peak systolic pressure over 10–15 consecutive cardiac cycles.

### 2.3 Echocardiographic assessment of right ventricular wall thickness

Transthoracic echocardiography was performed using a high-resolution imaging system (Vevo 2100, VisualSonics) equipped with a 30–40 MHz probe. Mice were lightly anesthetized with 1%–1.5% isoflurane to maintain heart rates at 450–550 bpm, ensuring light anesthesia during imaging. Right ventricular anterior wall thickness was measured in diastole from the parasternal short-axis view at the mid-papillary level. Three to five consecutive cardiac cycles were averaged for each mouse.

### 2.4 Right ventricular hypertrophy (fulton index)

Following hemodynamic measurements, mice were euthanized by CO_2_ inhalation (gradual fill rate of 20%–30% chamber volume/min), followed by cervical dislocation to ensure death. Hearts were excised and dissected to separate the right ventricle (RV) from the left ventricle plus interventricular septum (LV + S). Each portion was blotted dry and weighed. The Fulton index was calculated as RV/(LV + S).

### 2.5 Histology and morphometric analysis of pulmonary vascular remodeling

The pulmonary vasculature was perfused via the right ventricle with PBS to remove blood, followed by inflation–fixation of the lungs via the trachea with 4% paraformaldehyde at a constant pressure of 25 cm H_2_O for 10 min. Lungs were removed, fixed overnight, paraffin-embedded, and sectioned at 4 µm thickness. Hematoxylin–eosin (HE) staining was performed using standard protocols. Pulmonary vascular remodeling was quantified in small pulmonary arteries (external diameter 25–100 µm) that were circular or nearly circular in cross-section. All image analyses were performed by investigators blinded to group allocation.

Animal welfare was ensured by daily monitoring (twice daily during early hypoxia/treatment), with predefined humane endpoints (>20% weight loss, severe dyspnea, moribund state, etc.) and immediate euthanasia when reached; adverse events, exclusions (failed catheterization or non-diagnostic echocardiography), and survival numbers are reported. Sample size was determined *a priori* for 80% power allowing 10%–15% attrition, with details provided in the Statistics section.

### 2.6 Cell culture

Human Pulmonary Artery Smooth Muscle Cells (HPASMCs) were purchased from Lonza (Basel, Switzerland) and cultured in smooth muscle growth medium (SmGM™-2; Lonza) containing the manufacturer’s recommended growth factors and supplemented with fetal bovine serum (FBS) to a final concentration of 10%. Cells were maintained at 37 °C in a humidified atmosphere containing 5% CO_2_. According to the supplier, HPASMCs stained positive for smooth muscle α-actin and negative for von Willebrand factor (factor VIII). Cells between passages 4 and 8 were used for all experiments.

### 2.7 Construction and use of Ad-MELK

An adenovirus overexpressing human MELK (Ad-MELK) was generated with the AdEasy Vector System Kit (Agilent Technologies) following the manufacturer’s instructions. The coding sequence of human MELK was subcloned into the pCMV shuttle vector using primers containing restriction sites for BglII (AGATCT) and BamHI (GGATCC) (Primer F: ATGAAAGATTATGATGAACTTCTCAAATATTATG, Primer R: TACCTTGCAGCTAGATAGGATG). Recombinant adenoviruses were produced in HEK293 cells, purified using a commercial adenovirus purification kit (Adeno-X™ Maxi Purification Kit), and titrated to 1 × 10^11^ pfu/mL. For infection, viruses were added to cultured cells in serum- and antibiotic-free medium for 6 h, after which cells were switched to fresh complete medium and cultured for an additional 18 h before downstream assays. The control adenovirus carried the same backbone and promoter but lacked the MELK coding sequence.

### 2.8 Quantitative reverse transcription polymerase chain reaction (RT-qPCR)

Total RNA was extracted from HPASMCs by TRIzol (Ambion, United States). RNA reverse transcription to generate cDNA was conducted with the reverse transcription reagent Evo M-MLV RT Master Mix (Agbio, Hunan, China). Gene expression was evaluated by a Light Cycler 480 real-time PCR instrument (Roche, Indianapolis, IN, United States). The reaction conditions were set using a fluorescent quantitative PCR kit (SYBR Green Premix, Agbio, Hunan, China). The results were analyzed with the 2−△△Ct method and normalized to β-actin gene expression. The primers are listed in Table 1.

**TABLE 1:** Primers used for RT-PCR in this study.

Gene	Forward primers (5′–3′)	Reverse primers (5′–3′)
PCNA (human)	CCTGCTGGGATATTAGCTCCA	CAGCGGTAGGTGTCGAAGC
Cyclin D(human)	GCTGCGAAGTGGAAACCATC	CCTCCTTCTGCACACATTTGAA
Melk (human)	TCTCCCAGTAGCATTCTGCTT	TGATCCAGGGATGGTTCAATAGA
Cyr61 (human)	CTCGCCTTAGTCGTCACCC	CGCCGAAGTTGCATTCCAG
Ctgf (human)	CAGCATGGACGTTCGTCTG	AACCACGGTTTGGTCCTTGG
Birc5 (human)	AGGACCACCGCATCTCTACAT	AAGTCTGGCTCGTTCTCAGTG
Cyclin E (human)	AAGGAGCGGGACACCATGA	ACGGTCACGTTTGCCTTCC
YAP (human)	TAGCCCTGCGTAGCCAGTTA	TCATGCTTAGTCCACTGTCTGT
TAZ (human)	CAGCCAAATCTCGTGATGAATC	GGTTCTGCTGGCTCAGGGT
LATS1 (human)	AATTTGGGACGCATCATAAAGCC	TCGTCGAGGATCTTGGTAACTC
LATS2 (human)	ACTTTTCCTGCCACGACTTATTC	GATGGCTGTTTTAACCCCTCA
TAGLN (human)	AGTGCAGTCCAAAATCGAGAAG	CTTGCTCAGAATCACGCCAT
CNN1 (human)	CTGTCAGCCGAGGTTAAGAAC	GAGGCCGTCCATGAAGTTGTT
ACTA2 (human)	AAAAGACAGCTACGTGGGTGA	GCCATGTTCTATCGGGTACTTC
GAPDH (human)	GGAGCGAGATCCCTCCAAAAT	GGCTGTTGTCATACTTCTCATGG

### 2.9 Protein extraction and western blot

Proteins were extracted from cells and tissues using RIPA lysis buffer with a protease inhibitor (Merck, Darmstadt, Germany) and quantified by BCA assay (Thermo Fisher Scientific, Waltham, MA). Proteins were resolved in 6%–12% SDS-PAGE gels and then transferred onto polyvinylidene fluoride membranes (Millipore, United States). The membranes were blocked with 5% non-fat milk for 1 h at room temperature and then incubated with primary antibodies overnight at 4 °C. The primary antibodies used were as follows: Melk (1:2000 dilution; 11403-1-AP), ACTA2 (1:20,000, 67735-1-Ig), CNN1 (1:2000 dilution; 24855-1-AP), SM22α (1:5000 dilution; 10493-1-AP), YAP1 (1:2000, 13584-1-AP), TAZ (1:2000 dilution; 66500-1-Ig), Alpha Tubulin (1:20000 dilution; 66031-1-Ig) (ProteinTech), PCNA (1:1000 dilution; ab29), Cyclin D1(1:1000 dilution; ab16663), GAPDH (1:500, ab8245) (Abcam), Phospho-YAP (Ser127) (1:1000 dilution; # 13008) (Cell Signaling Technology). After washing with TBST, the membranes were incubated with corresponding secondary antibodies. Signals were detected by the standard ECL kit (Thermo Fisher Scientific), and densitometry was evaluated with ImageJ_v1.8.0 software.

### 2.10 RNA-seq data re-analysis

Publicly available RNA-sequencing data from GSE144274 were re-analyzed to identify genes differentially expressed in PASMCs from patients with idiopathic pulmonary arterial hypertension (IPAH) and healthy controls. Raw sequencing reads were downloaded from the Gene Expression Omnibus (GEO) database and processed following standard pipelines. Quality control was performed using FastQC, and adapter trimming and low-quality read removal were conducted with Trimmomatic. Clean reads were aligned to the human reference genome (GRCh38) using STAR. Gene-level counts were obtained with featureCounts, and differential expression analysis was performed using the DESeq2 R package. Genes with an adjusted *P*-value <0.05 and |log_2_ fold-change| ≥ 1 were defined as differentially expressed. Multiple testing correction was applied using the Benjamini–Hochberg method. Functional enrichment analyses, including Kyoto Encyclopedia of Genes and Genomes (KEGG) analyses, were conducted using the clusterProfiler R package to assess biological processes and pathways associated with the differentially expressed genes.

### 2.11 Immunofluorescence staining

OCT-embedded lung tissues and paraformaldehyde-fixed cells were permeabilized with 0.1% Triton X-100, blocked with 5% bovine serum albumin (BSA) at room temperature, and then incubated with primary antibodies overnight at 4 °C. After being washed with PBS, the sections or cells were incubated with corresponding secondary antibodies. Nuclei were counterstained with 4′,6-diamidino-2-phenylindole (DAPI). The antibodies used in this study were as follows: anti-MELK (1:2000 dilution; 11403-1-AP, Proteintech) and anti-ACTA2 (1:20,000 dilution; 67735-1-Ig, Proteintech). Fluorescence images of cells and lung tissues were acquired using a confocal laser scanning microscope (LSM 980, Carl Zeiss, Oberkochen, Germany).

### 2.12 Cell proliferation assays

HPASMCs were seeded to reach approximately 50%–60% confluence at the time of infection. Cells were transduced with Ad-MELK or control adenovirus (Ad-Con) at a multiplicity of infection (MOI) of 20 for 4 h in Opti-MEM (Gibco) without serum or antibiotics. After transduction, the medium was replaced with smooth muscle growth medium containing 0.3% FBS, with or without PDGF-BB (20 ng/mL). For inhibitor studies, cells were pretreated with OTS167 (10 nM) or vehicle for 1 h before PDGF-BB stimulation and maintained in the presence of the inhibitor throughout the culture period. At 46 h post-transduction, EdU (10 μM) was added to the culture for a 2 h pulse. At 48 h post-transduction, cells were harvested for proliferation analysis: Cell counting–Cells were trypsinized and counted using an automated cell counter (Countess, Invitrogen) according to the manufacturer’s instructions. EdU incorporation assay–EdU incorporation was measured using the Click-iT™ EdU Proliferation Assay Kit (Thermo Fisher Scientific) according to the manufacturer’s instructions. Fluorescence intensity was quantified using a BMG POLARstar Omega microplate reader.

### 2.13 Wound healing assay

HPASMCs were either infected with control adenovirus (Ad-Con) or MELK-overexpressing adenovirus (Ad-Melk) (MOI = 20, 4 h in Opti-MEM; Gibco), or pretreated with vehicle or the MELK inhibitor OTS167 (10 nM; MedChemExpress) for 24 h. Following infection or inhibitor pretreatment, cells were seeded into 6-well plates and cultured to 90%–100% confluence. A linear wound was created using a sterile 200 µL pipette tip, and detached cells were removed by two PBS washes. Cells were then incubated in serum-free smooth muscle growth medium with or without PDGF-BB (20 ng/mL; PeproTech) for 24 h. Images were captured at 0 h and 24 h post-scratch using an inverted phase-contrast microscope (Nikon). Wound closure was quantified using ImageJ software and expressed as the percentage of residual wound area relative to the initial wound area.

### 2.14 Statistical analysis

All statistical analyses were conducted using GraphPad Prism software (version 9.0; GraphPad Software, La Jolla, CA, United States). Quantitative results are presented as mean ± standard deviation (SD). The Shapiro–Wilk test was used to assess data normality. For normally distributed data, differences between two groups were analyzed by a two-tailed unpaired Student’s t-test, and comparisons among three or more groups were performed using one-way or two-way analysis of variance (ANOVA) followed by Dunnett’s or Tukey’s *post hoc* test. For data not following a normal distribution, the Mann–Whitney U test was applied for two-group comparisons. *P* < 0.05 was considered statistically significant.

## 3 Results

### 3.1 MELK expression is upregulated in pulmonary arterial hypertension and PDGF-BB–stimulated smooth muscle cells

**FIGURE 1:**
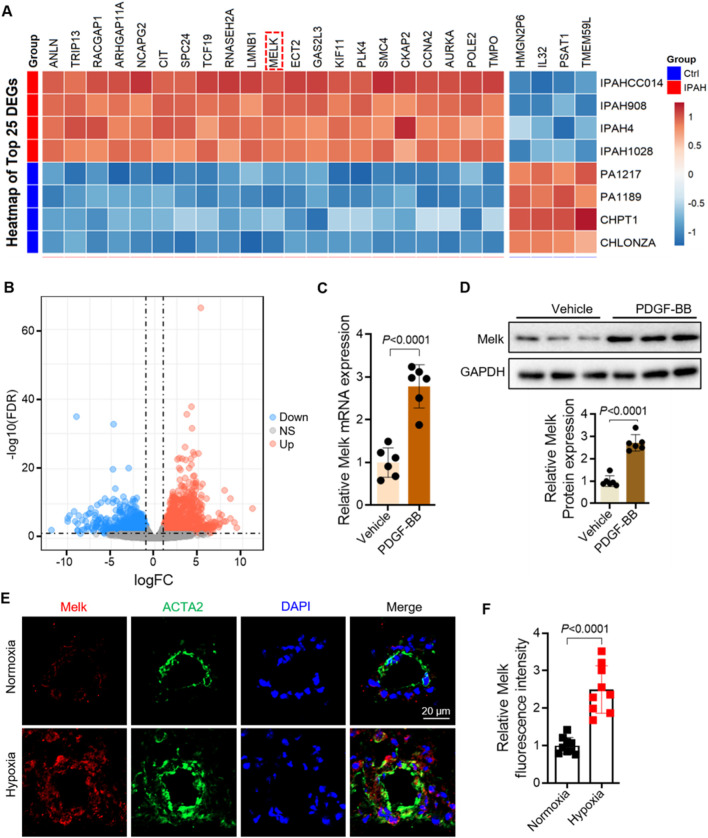
Maternal Embryonic Leucine Zipper Kinase (MELK) is significantly upregulated in pulmonary artery smooth muscle cells (PASMCs) of pulmonary arterial hypertension (PAH). **(A)** Heatmap of the GEO dataset GSE144274 showing the top 25 differentially expressed genes (DEGs) in PASMCs from patients with idiopathic pulmonary arterial hypertension (IPAH) and healthy controls. MELK is markedly upregulated in the IPAH group. **(B)** Volcano plot showing DEGs between PAH and control groups. **(C)** RT-qPCR analysis showing that human pulmonary artery smooth muscle cells (HPASMCs) treated with PDGF-BB (20 ng/mL, 48 h) exhibited a significant increase in Melk mRNA expression compared with vehicle-treated cells (n = 6). **(D)** Western blot and quantitative analysis showing that PDGF-BB stimulation (20 ng/mL, 48 h) markedly increased MELK protein expression in HPASMCs (n = 6). **(E)** Representative immunofluorescence images showing enhanced co-localization of MELK (red) and the SMC marker ACTA2 (green) in the pulmonary arteries of Sugen/hypoxia (Su/H) mice exposed to chronic hypoxia, whereas normoxia control mice displayed minimal MELK expression in the vessel wall. **(F)** Quantification of MELK fluorescence intensity showing a significant increase in the Su/H + hypoxia group compared with the normoxia group (n = 9). Data are presented as mean ± SD. Two-tailed unpaired Student’s t-test for two-group comparisons **(C,D,F)**; FDR-adjusted thresholds for **(B)**. *P* < 0.05 was considered statistically significant.

To identify genes dysregulated in human PAH, we analyzed the publicly available RNA-seq dataset GSE144274, which profiled HPASMCs isolated from IPAH and healthy donors. We visualized the top 25 most differentially expressed genes (DEGs) between IPAH and control HPASMCs, among which MELK was prominently upregulated (Figure 1A). Heatmap analyses confirmed robust MELK elevation in diseased HPASMCs (Figure 1B). To validate these transcriptomic findings, primary HPASMCs were stimulated with PDGF-BB to mimic the proliferative phenotype characteristic of PAH. RT-qPCR demonstrated a marked increase in MELK mRNA expression following PDGF-BB stimulation, and Western blot analysis further confirmed a significant elevation in MELK protein levels (Figures 1C,D). Consistent with these *in vitro* and human data, immunofluorescence staining of lung tissues from Sugen/hypoxia (Su/H) mice exposed to chronic hypoxia revealed strong co-localization of MELK with the SMC marker ACTA2 in pulmonary arterial walls, whereas normoxia control mice exhibited weak MELK staining (Figure 1E). Quantitative analysis indicated that MELK fluorescence intensity was significantly higher in Su/H + hypoxia mice compared with normoxia controls (Figure 1F). Collectively, these results demonstrate that MELK is significantly upregulated in both HPASMCs and experimental PAH models, suggesting a conserved role of MELK in promoting pulmonary vascular remodeling.

### 3.2 MELK inhibition by OTS167 suppresses proliferation and migration of HPASMCs

**FIGURE 2:**
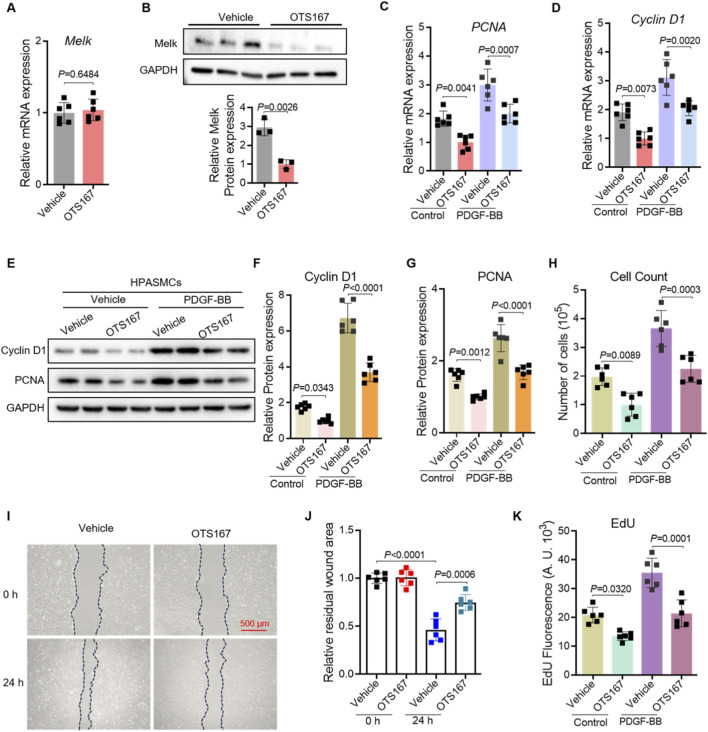
OTS167 inhibits MELK expression, suppressing proliferation and migration of HPASMCs. HPASMCs were treated with vehicle or the MELK inhibitor OTS167 (10 nM, 48 h) under basal conditions or following PDGF-BB stimulation (20 ng/mL, 48 h). **(A)** Quantitative PCR analysis of MELK transcript levels in HPASMCs treated with vehicle or the MELK inhibitor OTS167. Expression was normalized to a housekeeping gene and presented relative to vehicle. **(B)** Representative Western blot showing MELK protein in vehicle- and OTS167-treated HPASMCs, with GAPDH as a loading control (upper panel). Densitometric quantification of MELK protein normalized to GAPDH is shown (lower panel). **(C,D)** Relative mRNA expression of PCNA **(C)** and Cyclin D1 **(D)** determined by qRT-PCR. OTS167 significantly decreased the expression of both proliferation markers under basal and PDGF-BB–stimulated conditions (n = 6 independent experiments per group). **(E)** Representative immunoblots showing protein levels of PCNA and Cyclin D1; GAPDH served as a loading control. **(F,G)** Quantification of Cyclin D1 **(F)** and PCNA **(G)** protein expression normalized to GAPDH. **(H)** Cell counts showing reduced total cell numbers following OTS167 treatment. **(I)** Representative scratch wound healing images at 0 h and 24 h for HPASMCs treated with vehicle or OTS167. Dashed blue lines indicate wound edges. Scale bar = 500 μm. **(J)** Quantification of residual wound area at 24 h, showing reduced migration with OTS167 treatment. **(K)** EdU incorporation assay showing decreased DNA synthesis in OTS167-treated HPASMCs under basal and PDGF-BB–stimulated conditions. Data are presented as mean ± SD. n = 6 per group. Two-tailed unpaired Student’s t-test for two-group comparisons **(A,B)**, Two-way ANOVA followed by Tukey’s *post hoc* test was used **(C,D,F–H,J,K)**. *P* < 0.05 was considered statistically significant.

To explore the role of Melk in regulating smooth muscle cell proliferation and migration, we first assessed its expression following treatment with the Melk inhibitor OTS167. Quantitative PCR revealed that OTS167 did not affect Melk mRNA levels, while Western blotting demonstrated a marked decrease in Melk protein abundance, indicating that OTS167 effectively suppresses Melk at the post-transcriptional level (Figures 2A,B). We then examined the impact of Melk inhibition on proliferative activity of HPASMCs. Stimulation with PDGF-BB robustly increased the expression of the proliferation markers PCNA and Cyclin D1 at both the mRNA and protein levels, whereas treatment with OTS167 substantially attenuated these increases (Figures 2C–G). Consistently, cell counting assays revealed that OTS167 reduced the overall number of proliferating HPASMCs under PDGF-BB stimulation, further supporting an essential role of Melk in smooth muscle cell growth (Figure 2H). Because abnormal migration of smooth muscle cells also contributes to vascular remodeling, we evaluated the effect of OTS167 on HPASMC motility. In wound healing assays, PDGF-BB promoted rapid wound closure, whereas OTS167 markedly inhibited this migratory response, leaving a larger residual wound area after 24 h (Figures 2I,J). In parallel, EdU incorporation assays demonstrated that PDGF-BB strongly enhanced DNA synthesis, while OTS167 significantly suppressed EdU incorporation, confirming that Melk inhibition reduces cell cycle progression and DNA replication (Figure 2K). Together, these findings demonstrate that pharmacological inhibition of Melk with OTS167 effectively downregulates proliferative signaling and restrains both proliferation and migration of HPASMCs in response to PDGF-BB, highlighting Melk as a critical regulator of pathological vascular smooth muscle cell activation.

### 3.3 MELK overexpression promotes proliferation and migration of HPASMCs

**FIGURE 3:**
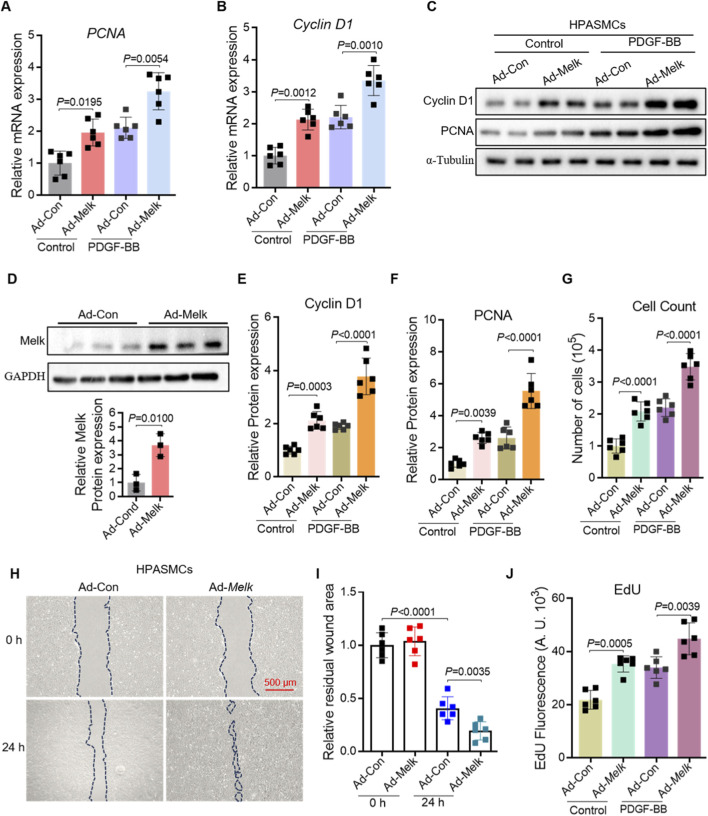
MELK overexpression promotes proliferation and migration of HPASMCs. HPASMCs were infected with control adenovirus (Ad-Con) or MELK-overexpressing adenovirus (Ad-Melk) under basal conditions or PDGF-BB stimulation (20 ng/mL, 48 h). **(A)** Relative mRNA expression of PCNA determined by qRT-PCR. MELK overexpression significantly increased PCNA mRNA levels under both basal and PDGF-BB-stimulated conditions. **(B)** Relative mRNA expression of Cyclin D1 determined by qRT-PCR, showing similar upregulation with MELK overexpression. **(C)** Representative immunoblots showing protein expression of PCNA and Cyclin D1 in HPASMCs, with α-Tubulin as a loading control. **(D)** Western blot showing MELK protein in Ad-Con vs. Ad-Melk cells with GAPDH as loading control. Lower panel: densitometric quantification of MELK normalized to GAPDH and expressed relative to Ad-Con. **(E,F)** Quantification of Cyclin D1 **(E)** and PCNA **(F)** protein expression normalized to α-Tubulin. MELK overexpression significantly increased protein levels under both basal and PDGF-BB-stimulated conditions. **(G)** Cell count assay showing increased total cell numbers following MELK overexpression. **(H)** Representative images of scratch wound healing assays at 0 h and 24 h. Dashed blue lines indicate wound edges. Scale bar, 500 μm. **(I)** Quantification of relative residual wound area showing that MELK overexpression enhanced HPASMC migration. **(J)** EdU incorporation assay showing that MELK overexpression increased DNA synthesis in HPASMCs. Data are presented as mean ± SD, n = 6 per group. Two-tailed unpaired Student’s t-test for two-group comparisons **(D)**. Two-way ANOVA followed by Tukey’s *post hoc* test was used **(A,B,E–G,I,J)**. *P* < 0.05 was considered statistically significant.

To further determine the functional role of Melk in HPASMCs, we performed gain-of-function experiments using adenoviral overexpression. Quantitative PCR analysis showed that Melk overexpression enhanced the expression of the proliferation markers PCNA and Cyclin D1 under basal conditions, and further potentiated their induction by PDGF-BB (Figures 3A,B). Western blot analysis confirmed that both Cyclin D1 and PCNA proteins were elevated in Melk-overexpressing cells compared with controls (Figure 3C). Consistently, direct measurement of Melk expression confirmed robust protein upregulation in Ad-Melk–transduced HPASMCs (Figure 3D). Densitometric quantification demonstrated that Melk overexpression significantly increased Cyclin D1 and PCNA protein levels in both basal and PDGF-BB–stimulated conditions (Figures 3E,F). In line with these molecular changes, cell counting assays revealed that Melk overexpression markedly promoted HPASMC proliferation (Figure 3G). Since vascular remodeling also involves enhanced migratory capacity of smooth muscle cells, we next assessed wound healing activity. In scratch assays, Ad-Melk–infected HPASMCs exhibited accelerated wound closure compared with control cells, both under resting and PDGF-BB–stimulated conditions (Figures 3H,I). Moreover, EdU incorporation assays demonstrated that Melk overexpression significantly enhanced DNA synthesis, indicating a strong pro-proliferative effect (Figure 3J). Together, these results demonstrate that Melk overexpression promotes HPASMC proliferation and migration by enhancing the expression of proliferation-associated genes and proteins, accelerating wound closure, and increasing DNA synthesis, thereby highlighting Melk as a key driver of pulmonary vascular smooth muscle cell activation.

### 3.4 MELK inhibition delays the phenotypic switch of HPASMCs from contractile to synthetic state

**FIGURE 4:**
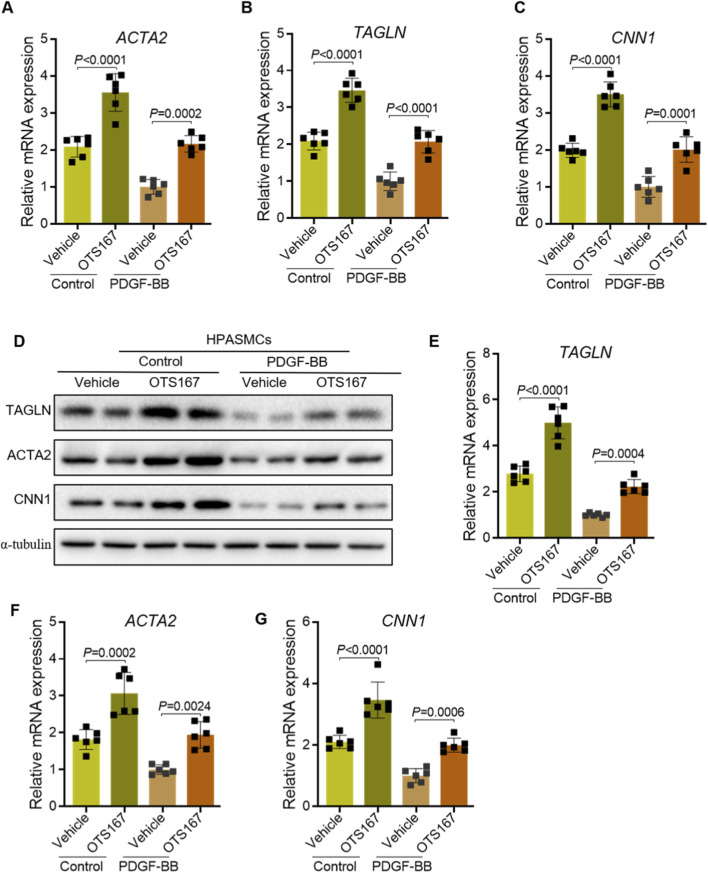
Inhibition of MELK delays the phenotypic switch of HPASMCs from a contractile to a synthetic phenotype. HPASMCs were treated with vehicle or OTS167 (10 nM, 48 h) under basal conditions or PDGF-BB stimulation (20 ng/mL, 48 h). **(A–C)** Relative mRNA expression of ACTA2 **(A)**, TAGLN **(B)**, and CNN1 **(C)** measured by qRT-PCR. OTS167 treatment significantly attenuated the PDGF-BB–induced downregulation of these contractile markers. **(D)** Representative immunoblots showing protein expression of ACTA2, CNN1, and SM22α in HPASMCs, with α-tubulin as a loading control. **(E–G)** Quantification of TAGLN **(E)**, ACTA2 **(F)**, and CNN1 **(G)** protein levels normalized to α-tubulin. OTS167 treatment preserved the expression of contractile markers under both basal and PDGF-BB-stimulated conditions. Data are presented as mean ± SD, n = 6 per group. Two-way ANOVA followed by Tukey’s *post hoc* test was used. *P* < 0.05 was considered statistically significant.

Quantitative real-time PCR analysis demonstrated that OTS167 treatment significantly attenuated the PDGF-BB–induced downregulation of the contractile markers ACTA2 (Figure 4A), TAGLN (Figure 4B), and CNN1 (Figure 4C). Consistent with these transcriptional changes, immunoblot analysis revealed that OTS167 preserved protein expression of ACTA2, CNN1, and SM22α under both basal and PDGF-BB–stimulated conditions (Figure 4D). Densitometric quantification confirmed that OTS167 markedly prevented the loss of TAGLN (Figure 4E), ACTA2 (Figure 4F), and CNN1 (Figure 4G) protein expression, indicating that MELK inhibition helps maintain the contractile phenotype. Together, these findings suggest that pharmacological inhibition of MELK by OTS167 mitigates the phenotypic switch of HPASMCs from a contractile to a synthetic state, even under strong mitogenic stimulation.

### 3.5 MELK promotes activation of the Hippo–YAP/TAZ signaling pathway in HPASMCs

**FIGURE 5:**
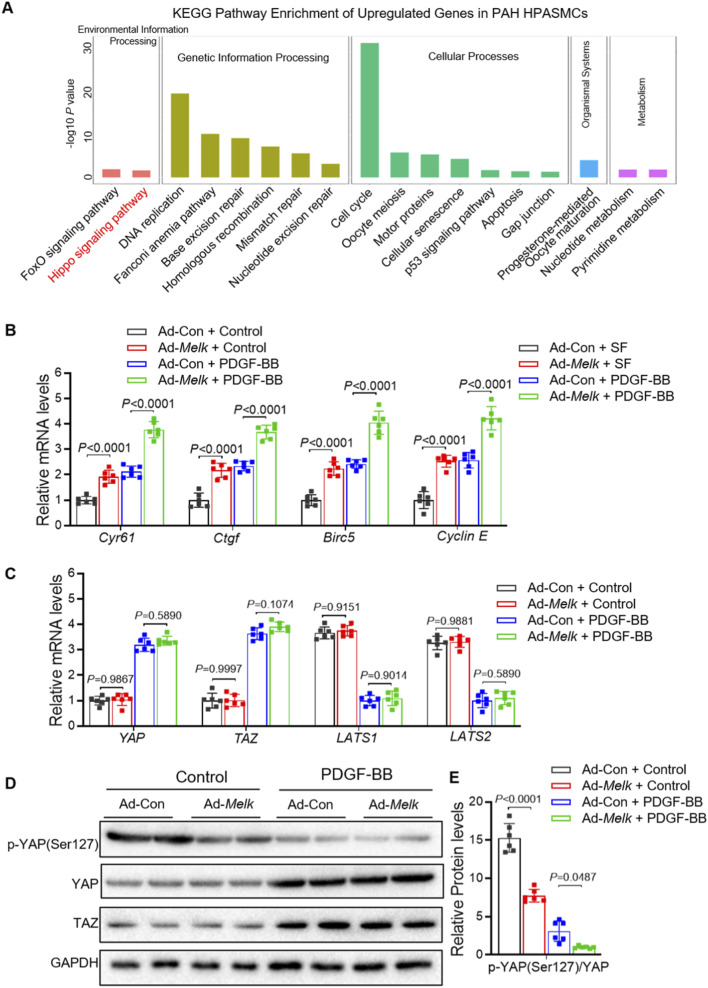
MELK promotes activation of the Hippo–YAP/TAZ signaling pathway in HPASMCs. **(A)** KEGG enrichment analysis of upregulated genes from RNA-seq dataset GSE144274 identified in HPASMCs derived from IPAH patients compared with healthy controls. For B-E, HPASMCs were infected with control adenovirus (Ad-Con) or MELK-overexpressing adenovirus (Ad-Melk) under basal conditions or PDGF-BB stimulation (20 ng/mL, 48 h). **(B)** Relative mRNA expression of YAP/TAZ downstream target genes (CYR61, CTGF, Birc5, and Cyclin **(E)** measured by qRT-PCR. MELK overexpression significantly upregulated these target genes under both basal and PDGF-BB-stimulated conditions. **(C)** Relative mRNA expression of upstream Hippo pathway core components (YAP, TAZ, LATS1, and LATS2) measured by qRT-PCR, showing no significant changes upon MELK overexpression. **(D)** Representative immunoblots showing phosphorylated YAP (p-YAP Ser127), total YAP, and TAZ protein levels in HPASMCs, with GAPDH as a loading control. **(E)** Quantification of p-YAP (Ser127) relative to total YAP, indicating that MELK overexpression reduced YAP phosphorylation under PDGF-BB stimulation, consistent with YAP/TAZ pathway activation. Data are presented as mean ± SD, n = 6 per group. Two-way ANOVA followed by Tukey’s *post hoc* test was used. *P* < 0.05 was considered statistically significant.

**FIGURE 6:**
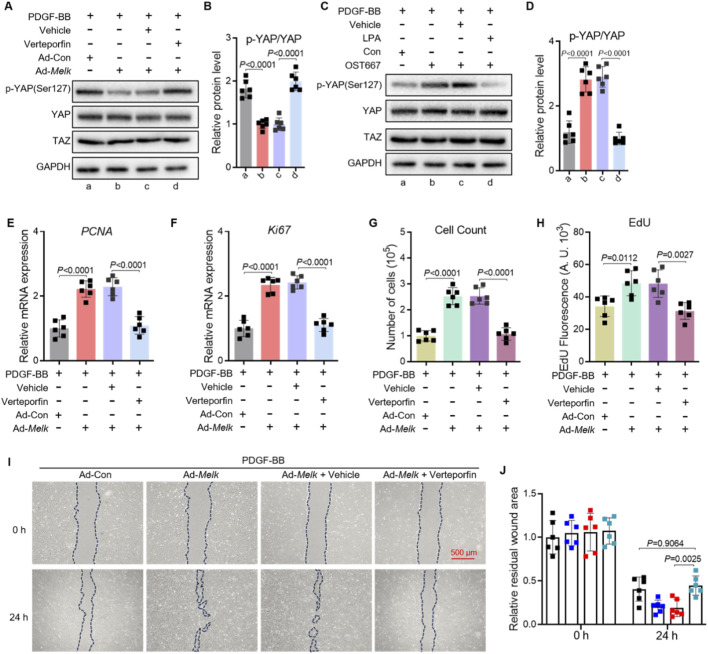
Hippo–YAP/TAZ signaling mediates MELK-induced proliferation and migration of PASMCs. **(A,B)** Representative immunoblot **(A)** and quantification **(B)** of phosphorylated YAP (p-YAP Ser127) and total YAP in PASMCs infected with control adenovirus (Ad-Con) or MELK-overexpressing adenovirus (Ad-MELK) and stimulated with PDGF-BB (20 ng/mL) for 48 h in the presence or absence of Verteporfin (1 μM). GAPDH served as a loading control. **(C,D)** Representative immunoblot **(C)** and quantification **(D)** of p-YAP and total YAP in PASMCs treated with PDGF-BB (20 ng/mL, 24 h) plus vehicle, LPA (1 μM), or OTS167 (10 nM). **(E,F)** Relative mRNA expression of PCNA **(E)** and Ki67 **(F)** measured by qRT-PCR after 48 h of treatment as in **(A)**. **(G)** Cell count assay after 48 h showing that Verteporfin reduced MELK-induced PASMC proliferation under PDGF-BB stimulation. **(H)** EdU incorporation assay after 48 h showing that Verteporfin decreased DNA synthesis in MELK-overexpressing PASMCs. **(I)** Representative images of scratch wound healing assays at 0 h and 24 h with dashed lines indicating wound edges. Scale bar, 500 μm. **(J)** Quantification of residual wound area at 24 h showing that Verteporfin attenuated MELK-induced PASMC migration. Data are mean ± SEM; n = 6 per group. One-way ANOVA with Tukey’s *post hoc* test; *P* < 0.05.

KEGG pathway enrichment analysis of upregulated genes from the HPASMC RNA-seq dataset (GSE144274) showed significant enrichment in Hippo signaling, cell cycle, and DNA repair–related pathways (Figure 6A). Among these, the Hippo pathway was one of the most prominently activated signaling cascades, suggesting its potential involvement in PAH pathogenesis. Given the critical role of the Hippo–YAP/TAZ signaling pathway in regulating cell proliferation, migration, and differentiation (Ma et al., 2019), its sustained activation contributes to pulmonary vascular remodeling in PAH (Kudryashova et al., 2016; Wang et al., 2019; Kudryashova et al., 2022). We sought to determine whether MELK influences this pathway as a potential mechanism underlying its effects on HPASMCs proliferation and migration. HPASMCs were infected with control adenovirus (Ad-Con) or MELK-overexpressing adenovirus (Ad-Melk) and cultured under basal conditions or with PDGF-BB stimulation (20 ng/mL, 24 h). Quantitative real-time PCR analysis revealed that MELK overexpression markedly increased mRNA levels of multiple YAP/TAZ downstream target genes, including Cyr61, Ctgf, Birc5, and Cyclin E, under both basal and PDGF-BB–stimulated conditions (Figure 5A). In contrast, MELK overexpression had no significant effect on the mRNA expression of upstream Hippo pathway core components (YAP, TAZ, LATS1, and LATS2) (Figure 5B). Immunoblot analysis showed that MELK overexpression reduced phosphorylation of YAP at Ser127 without altering total YAP or TAZ protein levels (Figure 5C). Densitometric quantification confirmed that MELK overexpression significantly decreased the p-YAP(Ser127)/total YAP ratio in PDGF-BB–stimulated cells (Figures 5D,E), consistent with activation of the YAP/TAZ pathway.

These findings indicate that MELK promotes Hippo–YAP/TAZ pathway activation by reducing inhibitory phosphorylation of YAP, thereby enhancing transcriptional activation of its downstream target genes. This mechanism may contribute to MELK-driven HPASMCs proliferation and migration.

### 3.6 Hippo–YAP/TAZ signaling mediates MELK-induced PASMC proliferation and migration

To determine whether the Hippo–YAP/TAZ pathway is required for MELK-induced proliferative and migratory responses in pulmonary artery smooth muscle cells (PASMCs), we performed YAP inhibition experiments using verteporfin. PASMCs were infected with control adenovirus (Ad-Con) or MELK-overexpressing adenovirus (Ad-Melk) and stimulated with PDGF-BB (20 ng/mL, 24 h) in the presence or absence of verteporfin (1 μM, 24 h). Western blot analysis demonstrated that MELK overexpression reduced YAP phosphorylation at Ser127, whereas verteporfin restored p-YAP(Ser127) levels (Figure 6A). Quantification confirmed that verteporfin significantly reversed MELK-induced suppression of YAP phosphorylation (Figure 6B). Similarly, in PASMCs treated with PDGF-BB and LPA (1 μM) or the MELK inhibitor OTS167 (10 nM), YAP phosphorylation levels were increased compared with MELK-overexpressing cells without inhibitor treatment (Figures 6C,D). qRT-PCR analysis revealed that MELK overexpression upregulated mRNA levels of proliferation markers PCNA and Ki67, which were markedly reduced by verteporfin under PDGF-BB stimulation (Figures 6E,F). Consistently, cell counting assays showed that verteporfin attenuated MELK-induced PASMC proliferation (Figure 6G), and EdU incorporation assays demonstrated decreased DNA synthesis upon verteporfin treatment (Figure 6H). Wound healing assays indicated that MELK overexpression enhanced PASMC migration, while verteporfin markedly suppressed this effect under PDGF-BB stimulation (Figure 6I). Quantification of the residual wound area at 24 h confirmed significant inhibition of MELK-induced migratory capacity by verteporfin (Figure 6J).

Collectively, these findings demonstrate that MELK promotes PASMC proliferation and migration at least in part through activation of the Hippo–YAP/TAZ signaling pathway, and that pharmacological inhibition of YAP with verteporfin effectively blunts these pathological responses.

### 3.7 OTS167 treatment attenuates pulmonary hypertension and vascular remodeling in Su/H mice

**FIGURE 7:**
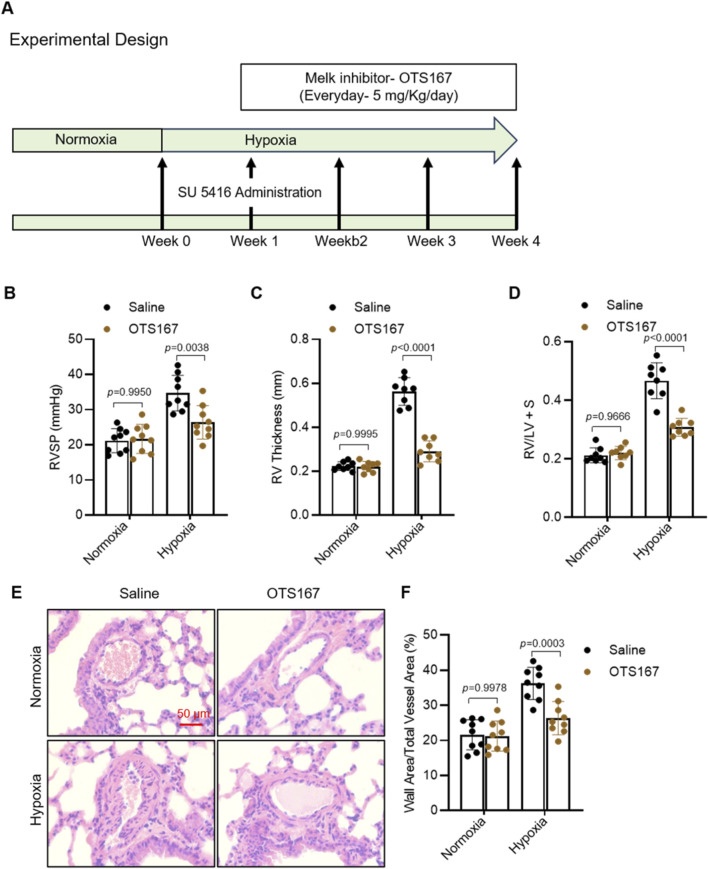
Effects of MELK inhibition by OTS-167 on PAH and vascular remodeling in Sugen/hypoxia (Su/H) mice. **(A)** Experimental design. Su/H mice were generated by intraperitoneal (IP) injection of Sugen 5416 (20 mg/kg, once weekly for 3 weeks) and exposure to 10% O_2_ for 4 weeks. Starting at the end of week 1, mice received daily IP injections of saline or the MELK inhibitor OTS-167 (5 mg/kg) for 3 weeks. **(B)** Right ventricular systolic pressure (RVSP) measured by right heart catheterization at week 4 (n = 9 per group). **(C)** Right ventricular (RV) free wall thickness assessed by echocardiography (Vevo3100) (n = 9 per group). **(D)** Fulton index [RV/(LV + septum)] determined by postmortem analysis at week 4 (n = 9 per group). **(E)** Representative hematoxylin and eosin (H&E)–stained lung sections showing pulmonary artery (PA) wall morphology (scale bars = 50 µm). **(F)** Quantification of PA vascular remodeling, expressed as percentage of vessels with wall thickness >50% of external diameter (n = 9 per group). Data are presented as mean ± SD. Two-way ANOVA followed by Tukey’s *post hoc* test was used. *P* < 0.05 was considered statistically significant.

To investigate the role of MELK inhibition in pulmonary hypertension, we employed a Sugen/hypoxia (Su/H) mouse model and treated mice with the MELK inhibitor OTS167 (5 mg/kg, IP daily) or saline starting at the end of week 1 for 3 weeks (Figure 7A). Hemodynamic assessment at week 4 revealed that Su/H mice exhibited a marked increase in right ventricular systolic pressure (RVSP) compared with normoxic controls (Figure 7B). OTS167 treatment reduced RVSP in Su/H mice, whereas no difference was observed in normoxic animals. Echocardiographic measurements demonstrated a significant increase in right ventricular (RV) wall thickness in Su/H mice, which was markedly attenuated by OTS167 (Figure 7C). Postmortem analysis of RV hypertrophy using the Fulton index confirmed that OTS167 reduced RV hypertrophy in Su/H mice (Figure 7D), with no effect in normoxic controls. Histological analysis of hematoxylin and eosin (H&E)-stained lung sections revealed pronounced pulmonary vascular remodeling in Su/H mice, characterized by increased medial wall thickness of small pulmonary arteries (Figure 7E). Quantitative morphometric analysis showed a higher percentage of wall area relative to total vessel area in Su/H mice compared with normoxia, which was reduced by OTS167 treatment (Figure 7F). OTS167 did not affect vascular morphology in normoxic mice. Collectively, these results demonstrate that pharmacological inhibition of MELK by OTS167 effectively attenuates pulmonary hypertension, RV hypertrophy, and pulmonary vascular remodeling in Su/H mice.

## 4 Discussion

In this study, we identified MELK as a previously unrecognized driver of pathological PASMC remodeling in PAH. Using both overexpression and pharmacological inhibition strategies, we demonstrated that MELK promotes PASMC proliferation, migration, and phenotypic switching from a contractile to a synthetic phenotype, accompanied by activation of the Hippo–YAP/TAZ signaling pathway. These findings suggest MELK as a mechanistic link between upstream mitogenic stimuli, such as platelet-derived growth factor-BB (PDGF-BB), and the transcriptional programs that govern vascular remodeling in PAH.

MELK has been implicated in cell cycle regulation, DNA damage repair, apoptosis, and stem cell maintenance in multiple malignancies and regenerative processes (Chlenski et al., 2019; Nakano et al., 2011; Wang et al., 2016). Its expression is typically low in normal differentiated tissues but becomes highly upregulated in proliferative contexts, including embryonic development, wound healing, and tumorigenesis (Szymanski et al., 2023; Jiang and Zhang, 2013; Su et al., 2025). Our results demonstrate that MELK overexpression in PASMCs markedly enhances the expression of proliferation markers (PCNA, Cyclin D1), increases DNA synthesis, and accelerates cell migration, whereas MELK inhibition by OTS167 produces the opposite effects. Importantly, these responses were evident both under basal conditions and in the presence of PDGF-BB, a major growth factor implicated in PAH pathogenesis (Zhao et al., 2014).

The Hippo–YAP/TAZ pathway is a central regulator of organ size, tissue regeneration, and tumorigenesis, and its dysregulation contributes to pulmonary vascular remodeling (Kudryashova et al., 2016; Wang et al., 2019; Kudryashova et al., 2022; Tang et al., 2022; Zuo et al., 2021). YAP/TAZ activity is primarily controlled by phosphorylation through the upstream kinases MST1/2 and LATS1/2, which retain YAP/TAZ in the cytoplasm and promote their degradation (Moya and Halder, 2019). When Hippo signaling is inhibited, dephosphorylated YAP/TAZ translocate into the nucleus and drive the transcription of genes involved in proliferation (CYR61, CTGF, Birc5, Cyclin E), migration, and extracellular matrix production (Piccolo et al., 2014). Our data show that MELK overexpression reduces the inhibitory phosphorylation of YAP at Ser127, leading to enhanced expression of canonical YAP/TAZ target genes. Conversely, MELK inhibition restores p-YAP levels, thereby suppressing YAP/TAZ-driven transcription. These findings place MELK upstream of YAP/TAZ activation in PASMCs.

Although the precise molecular intermediates remain undefined, several plausible mechanisms may explain how MELK regulates Hippo–YAP/TAZ activity in PASMCs. Our data show that MELK overexpression reduces YAP phosphorylation and increases YAP/TAZ transcriptional output, but the upstream events remain unclear. As a serine/threonine kinase with diverse substrates, MELK may modulate Hippo–YAP/TAZ through multiple routes. Previous studies in tumor cells have shown that MELK can phosphorylate downstream regulatory proteins such as EZH2 and ASK1, thereby altering the activity of major signaling pathways (Li et al., 2019; Seong et al., 2017). This suggests that MELK may also act on key regulators of the Hippo pathway. Specifically, MELK may inhibit the upstream MST1/2–LATS1/2 kinase cascade, either by directly phosphorylating LATS regulators or by interfering with adaptor proteins such as MOB1, thereby reducing YAP phosphorylation and promoting its nuclear translocation. YAP/TAZ activity is tightly regulated by cytoskeletal tension (Kim et al., 2016). Although direct regulation of actin-associated proteins by MELK has not been reported, evidence from colorectal cancer cells indicates that MELK knockdown significantly reduces RhoA activity without affecting Rac1 or Cdc42 (Du et al., 2014), suggesting that MELK may influence cytoskeletal organization through the RhoA pathway and thereby modulate YAP/TAZ activation. In addition, MELK has been shown in multiple cancers to activate PI3K–Akt–mTOR signaling, and these mitogenic pathways can indirectly promote YAP/TAZ activity. Thus, MELK might cooperate with pathways such as PI3K–Akt–mTOR and ERK–MAPK, both of which are activated by PDGF-BB and known to enhance YAP/TAZ function (Xu et al., 2020). MELK has also been linked to epigenetic regulation through phosphorylation-mediated stabilization of EZH2, resulting in increased histone H3K27 trimethylation (H3K27me3) at target promoters (Yu et al., 2024). Because EZH2 can physically interact with YAP/TAZ to enhance TEAD-dependent transcription (Lo Sardo et al., 2021), MELK-driven EZH2 activation may indirectly amplify YAP/TAZ signaling. These proposed mechanisms remain speculative and require further validation through biochemical assays, phosphoproteomic profiling, and genetic perturbation of candidate intermediates in PASMCs. Such studies will be essential to clarify whether MELK directly phosphorylates Hippo pathway components, influences cytoskeletal organization, or acts through parallel signaling and epigenetic mechanisms to regulate YAP/TAZ activity in PAH. Future work integrating phosphoproteomics, and high-resolution cytoskeletal imaging will be critical to delineate the predominant regulatory route and assess its therapeutic relevance in pulmonary vascular remodeling.

One of the most striking findings of our study is that MELK inhibition preserves the contractile phenotype of PASMCs, as evidenced by sustained expression of contractile markers ACTA2, TAGLN, and CNN1 even under PDGF-BB stimulation. Phenotypic switching of PASMCs—from a quiescent, contractile state to a synthetic, proliferative state—is a hallmark of vascular remodeling in PAH (Kim et al., 2025). This transition facilitates excessive cell proliferation, extracellular matrix deposition, and medial thickening, all of which contribute to elevated pulmonary vascular resistance (Kim et al., 2025). By attenuating this switch, MELK inhibition not only limits cell proliferation but also helps maintain vascular tone and compliance. This dual effect may provide a therapeutic advantage over strategies that target proliferation alone.

From a translational perspective, our findings support MELK as a promising therapeutic target in PAH. OTS167, the MELK inhibitor used in this study, has advanced to clinical trials for several cancers and exhibits high selectivity and potency (Stefka et al., 2016). The fact that OTS167 effectively suppressed PASMC proliferation, migration, and YAP/TAZ activation suggests its potential for repurposing in PAH. Moreover, the YAP inhibitor verteporfin, which is already FDA-approved for ocular neovascular diseases (Ziemssen and Heimann, 2012), similarly reversed MELK-driven pathological responses, indicating that dual blockade of MELK and YAP/TAZ could yield synergistic benefits. However, systemic inhibition of MELK or YAP/TAZ may have off-target effects, particularly in tissues with high regenerative activity, necessitating strategies to achieve pulmonary vascular selectivity.

OTS167 treatment attenuated pulmonary hypertension, right ventricular hypertrophy, and pulmonary vascular remodeling in Su/H mice, supporting the therapeutic potential of targeting MELK signaling in PAH. Several limitations warrant consideration. First, while our *in vitro* data are compelling, *in vivo* validation using established PAH models such as the Sugen/hypoxia rat or monocrotaline-induced PAH model is necessary to assess the therapeutic potential of MELK inhibition in a physiologically relevant context. Second, although OTS167 is widely used as a potent ATP-competitive MELK inhibitor (IC_50_ ≈ 0.4 nM) (Chung et al., 2012), accumulating evidence indicates that its effects are not exclusively MELK-dependent. Recent studies demonstrated that OTS167 can exert off-target effects on other kinases and signaling pathways. Matsuda et al. showed that OTS167 induces p21 expression via the FOXO1/3–p21 axis (Matsuda et al., 2017); Lin et al. reported that OTS167 retains cytotoxic activity even in MELK-deficient cells (Lin et al., 2017); and Eisfelder et al. revealed that OTS167 acts through a dual mechanism, inhibiting the MELK–eIF4B translational axis and directly suppressing FLT3 kinase activity (Eisfelder et al., 2021). These findings suggest that OTS167 should be regarded as a potent but not fully selective MELK inhibitor, and the observed *in vivo* benefits may partially involve MELK-independent mechanisms. Future studies employing more selective MELK inhibitors or genetic knockout models will be necessary to confirm the causal role of MELK in PAH pathogenesis. Finally, while we focused on Hippo–YAP/TAZ signaling, MELK may also intersect with other PAH-relevant pathways, including PI3K–Akt–mTOR, ERK1/2–MAPK, and STAT3 signaling (Liu et al., 2023; Tian et al., 2021; Bisserier et al., 2021). The potential contribution of MELK in other vascular cell types, such as pulmonary artery endothelial cells and adventitial fibroblasts, remains to be determined.

In summary, our findings reveal MELK as a key upstream regulator of Hippo–YAP/TAZ signaling and PASMC phenotypic modulation in PAH. By driving proliferation, migration, and contractile-to-synthetic switching, MELK contributes to pulmonary vascular remodeling. Pharmacological inhibition of MELK or downstream YAP/TAZ effectively reverses these pathological changes, highlighting the therapeutic potential of targeting the MELK–Hippo–YAP/TAZ axis as a potential therapeutic strategy. These results not only deepen our mechanistic understanding of PAH pathogenesis but also lay the groundwork for translational studies aimed at developing MELK-targeted interventions for this fatal disease.

## Data Availability

The raw data supporting the conclusions of this article will be made available by the authors, without undue reservation.

## References

[B1] Bisserier M. Katz M. G. Bueno-Beti C. Brojakowska A. Zhang S. Gubara S. (2021). Combination therapy with STAT3 inhibitor enhances SERCA2a-Induced BMPR2 expression and inhibits pulmonary arterial hypertension. Int. J. Mol. Sci. 22, 9105. 10.3390/ijms22179105 34502015 PMC8431626

[B2] Born E. Lipskaia L. Breau M. Houssaini A. Beaulieu D. Marcos E. (2023). Eliminating senescent cells can promote pulmonary hypertension development and progression. Circulation 147, 650–666. 10.1161/CIRCULATIONAHA.122.058794 36515093

[B3] Chlenski A. Park C. Dobratic M. Salwen H. R. Budke B. Park J.-H. (2019). Maternal embryonic leucine zipper kinase (MELK), a potential therapeutic target for neuroblastoma. Mol. Cancer Ther. 18, 507–516. 10.1158/1535-7163.MCT-18-0819 30674566 PMC6398941

[B4] Chung S. Suzuki H. Miyamoto T. Takamatsu N. Tatsuguchi A. Ueda K. (2012). Development of an orally-administrative MELK-targeting inhibitor that suppresses the growth of various types of human cancer. Oncotarget 3, 1629–1640. 10.18632/oncotarget.790 23283305 PMC3681500

[B5] Du T. Qu Y. Li J. Li H. Su L. Zhou Q. (2014). Maternal embryonic leucine zipper kinase enhances gastric cancer progression via the FAK/Paxillin pathway. Mol. Cancer 13, 100. 10.1186/1476-4598-13-100 24885567 PMC4113179

[B6] Eisfelder B. J. Saygin C. Wynne J. Colton M. W. Fischietti M. Beauchamp E. M. (2021). OTS167 blocks FLT3 translation and synergizes with FLT3 inhibitors in FLT3 mutant acute myeloid leukemia. Blood Cancer J. 11, 48. 10.1038/s41408-021-00433-3 33658483 PMC7930094

[B7] Humbert M. Kovacs G. Hoeper M. M. Badagliacca R. Berger R. M. F. Brida M. (2022). 2022 ESC/ERS guidelines for the diagnosis and treatment of pulmonary hypertension. Eur. Heart J. 43, 3618–3731. 10.1093/eurheartj/ehac237 36017548

[B8] Jiang P. Zhang D. (2013). Maternal embryonic leucine zipper kinase (MELK): a novel regulator in cell cycle control, embryonic development, and cancer. Int. J. Mol. Sci. 14, 21551–21560. 10.3390/ijms141121551 24185907 PMC3856021

[B9] Kiang K. M. Ahad L. Zhong X. Lu Q. R. (2024). Biomolecular condensates: hubs of Hippo-YAP/TAZ signaling in cancer. Trends Cell Biol. 34, 566–577. 10.1016/j.tcb.2024.04.009 38806345

[B10] Kim M. H. Kim J. Hong H. Lee S.-H. Lee J.-K. Jung E. (2016). Actin remodeling confers BRAF inhibitor resistance to melanoma cells through YAP/TAZ activation. EMBO J. 35, 462–478. 10.15252/embj.201592081 26668268 PMC4772854

[B11] Kim Y. Yeo Y. Kim M. Son Y.-W. Kim J. Kim K. L. (2025). A highly mobile adeno-associated virus targeting vascular smooth muscle cells for the treatment of pulmonary arterial hypertension. Nat. Biomed. Eng. 9, 1418–1436. 10.1038/s41551-025-01379-8 40301691

[B12] Kudryashova T. V. Goncharov D. A. Pena A. Kelly N. Vanderpool R. Baust J. (2016). HIPPO-Integrin-linked kinase cross-talk controls self-sustaining proliferation and survival in pulmonary hypertension. Am. J. Respir. Crit. Care Med. 194, 866–877. 10.1164/rccm.201510-2003OC 27119551 PMC5074651

[B13] Kudryashova T. V. Dabral S. Nayakanti S. Ray A. Goncharov D. A. Avolio T. (2022). Noncanonical HIPPO/MST signaling via BUB3 and FOXO drives pulmonary vascular cell growth and survival. Circ. Res. 130, 760–778. 10.1161/CIRCRESAHA.121.319100 35124974 PMC8897250

[B14] Lai Y.-C. Potoka K. C. Champion H. C. Mora A. L. Gladwin M. T. (2014). Pulmonary arterial hypertension: the clinical syndrome. Circ. Res. 115, 115–130. 10.1161/CIRCRESAHA.115.301146 24951762 PMC4096686

[B15] Li B. Yan J. Phyu T. Fan S. Chung T.-H. Mustafa N. (2019). MELK mediates the stability of EZH2 through site-specific phosphorylation in extranodal natural killer/T-cell lymphoma. Blood 134, 2046–2058. 10.1182/blood.2019000381 31434700

[B16] Lin A. Giuliano C. J. Sayles N. M. Sheltzer J. M. (2017). CRISPR/Cas9 mutagenesis invalidates a putative cancer dependency targeted in on-going clinical trials. Elife 6, e24179. 10.7554/eLife.24179 28337968 PMC5365317

[B17] Liu Y. Tang B.-L. Lu M.-L. Wang H.-X. (2023). Astragaloside IV improves pulmonary arterial hypertension by increasing the expression of CCN1 and activating the ERK1/2 pathway. J. Cell Mol. Med. 27, 622–633. 10.1111/jcmm.17681 36762748 PMC9983322

[B18] Lo Sardo F. Pulito C. Sacconi A. Korita E. Sudol M. Strano S. (2021). YAP/TAZ and EZH2 synergize to impair tumor suppressor activity of TGFBR2 in non-small cell lung cancer. Cancer Lett. 500, 51–63. 10.1016/j.canlet.2020.11.037 33296708

[B19] Ma S. Meng Z. Chen R. Guan K.-L. (2019). The hippo pathway: biology and pathophysiology. Annu. Rev. Biochem. 88, 577–604. 10.1146/annurev-biochem-013118-111829 30566373

[B20] Ma B. Cao Y. Qin J. Chen Z. Hu G. Li Q. (2023). Pulmonary artery smooth muscle cell phenotypic switching: a key event in the early stage of pulmonary artery hypertension. Drug Discov. Today 28, 103559. 10.1016/j.drudis.2023.103559 36958640

[B21] Matsuda T. Kato T. Kiyotani K. Tarhan Y. E. Saloura V. Chung S. (2017). p53-independent p21 induction by MELK inhibition. Oncotarget 8, 57938–57947. 10.18632/oncotarget.18488 28938528 PMC5601624

[B22] Mouraret N. Marcos E. Abid S. Gary-Bobo G. Saker M. Houssaini A. (2013). Activation of lung p53 by Nutlin-3a prevents and reverses experimental pulmonary hypertension. Circulation 127, 1664–1676. 10.1161/CIRCULATIONAHA.113.002434 23513067 PMC3989211

[B23] Moya I. M. Halder G. (2019). Hippo-YAP/TAZ signalling in organ regeneration and regenerative medicine. Nat. Rev. Mol. Cell Biol. 20, 211–226. 10.1038/s41580-018-0086-y 30546055

[B24] Nakano I. Joshi K. Visnyei K. Hu B. Watanabe M. Lam D. (2011). Siomycin A targets brain tumor stem cells partially through a MELK-mediated pathway. Neuro Oncol. 13, 622–634. 10.1093/neuonc/nor023 21558073 PMC3107094

[B25] Piccolo S. Dupont S. Cordenonsi M. (2014). The biology of YAP/TAZ: hippo signaling and beyond. Physiol. Rev. 94, 1287–1312. 10.1152/physrev.00005.2014 25287865

[B26] Seong H.-A. Manoharan R. Ha H. (2017). Zinc finger protein ZPR9 functions as an activator of AMPK-related serine/threonine kinase MPK38/MELK involved in ASK1/TGF-β/p53 signaling pathways. Sci. Rep. 7, 42502. 10.1038/srep42502 28195154 PMC5307367

[B27] Southgate L. Machado R. D. Gräf S. Morrell N. W. (2020). Molecular genetic framework underlying pulmonary arterial hypertension. Nat. Rev. Cardiol. 17, 85–95. 10.1038/s41569-019-0242-x 31406341

[B28] Stefka A. T. Park J. H. Matsuo Y. Chung S. Nakamura Y. Jakubowiak A. J. (2016). Anti-myeloma activity of MELK inhibitor OTS167: effects on drug-resistant myeloma cells and putative myeloma stem cell replenishment of malignant plasma cells. Blood Cancer J. 6, e460. 10.1038/bcj.2016.71 27540718 PMC5022182

[B29] Su P. Lu Q. Wang Y. Mou Y. Jin W. (2025). Targeting MELK in tumor cells and tumor microenvironment: from function and mechanism to therapeutic application. Clin. Transl. Oncol. 27, 887–900. 10.1007/s12094-024-03664-5 39187643

[B30] Szymanski L. Lewicki S. Markiewicz T. Cierniak S. Tassan J.-P. Kubiak J. Z. (2023). siRNA-Mediated MELK knockdown induces accelerated wound healing with increased collagen deposition. Int. J. Mol. Sci. 24, 1326. 10.3390/ijms24021326 36674843 PMC9861445

[B31] Tang Q. Li W. Zheng X. Ren L. Liu J. Li S. (2020). MELK is an oncogenic kinase essential for metastasis, mitotic progression, and programmed death in lung carcinoma. Signal Transduct. Target Ther. 5, 279. 10.1038/s41392-020-00288-3 33262323 PMC7708490

[B32] Tang W. Li M. Yangzhong X. Zhang X. Zu A. Hou Y. (2022). Hippo signaling pathway and respiratory diseases. Cell Death Discov. 8, 213. 10.1038/s41420-022-01020-6 35443749 PMC9021242

[B33] Tang B. Zhu J. Shi Y. Wang Y. Zhang X. Chen B. (2024). Tumor cell-intrinsic MELK enhanced CCL2-dependent immunosuppression to exacerbate hepatocarcinogenesis and confer resistance of HCC to radiotherapy. Mol. Cancer 23, 137. 10.1186/s12943-024-02049-0 38970074 PMC11225310

[B34] Thenappan T. Ormiston M. L. Ryan J. J. Archer S. L. (2018). Pulmonary arterial hypertension: pathogenesis and clinical management. BMJ 360, j5492. 10.1136/bmj.j5492 29540357 PMC6889979

[B35] Thompson A. A. R. Lawrie A. (2017). Targeting vascular remodeling to treat pulmonary arterial hypertension. Trends Mol. Med. 23, 31–45. 10.1016/j.molmed.2016.11.005 27989641

[B36] Tian H. Liu L. Wu Y. Wang R. Jiang Y. Hu R. (2021). Resistin-like molecule β acts as a mitogenic factor in hypoxic pulmonary hypertension via the Ca2+-dependent PI3K/Akt/mTOR and PKC/MAPK signaling pathways. Respir. Res. 22, 8. 10.1186/s12931-020-01598-4 33407472 PMC7789700

[B37] Wang Y. Begley M. Li Q. Huang H.-T. Lako A. Eck M. J. (2016). Mitotic MELK-eIF4B signaling controls protein synthesis and tumor cell survival. Proc. Natl. Acad. Sci. U. S. A. 113, 9810–9815. 10.1073/pnas.1606862113 27528663 PMC5024598

[B38] Wang Q. Shi W. Zhang Q. Feng W. Wang J. Zhai C. (2019). Inhibition of Siah2 ubiquitin ligase ameliorates monocrotaline-induced pulmonary arterial remodeling through inactivation of YAP. Life Sci. 242, 117159. 10.1016/j.lfs.2019.117159 31837334

[B39] Xu Q. Ge Q. Zhou Y. Yang B. Yang Q. Jiang S. (2020). MELK promotes endometrial carcinoma progression via activating mTOR signaling pathway. EBioMedicine 51, 102609. 10.1016/j.ebiom.2019.102609 31915116 PMC7000338

[B40] Yu H. Xu X. Zhu L. Chen S. He J. (2024). MELK aggravates lung adenocarcinoma by regulating EZH2 ubiquitination and H3K27me3 histone methylation of LATS2. J. Cell Mol. Med. 28, e18216. 10.1111/jcmm.18216 38652219 PMC11037405

[B41] Zhao Y. Lv W. Piao H. Chu X. Wang H. (2014). Role of platelet-derived growth factor-BB (PDGF-BB) in human pulmonary artery smooth muscle cell proliferation. J. Recept Signal Transduct. Res. 34, 254–260. 10.3109/10799893.2014.908915 24804810

[B42] Ziemssen F. Heimann H. (2012). Evaluation of verteporfin pharmakokinetics--redefining the need of photosensitizers in ophthalmology. Expert Opin. Drug Metab. Toxicol. 8, 1023–1041. 10.1517/17425255.2012.701617 22762303

[B43] Zuo W. Liu N. Zeng Y. Xiao Z. Wu K. Yang F. (2021). Luteolin ameliorates experimental pulmonary arterial hypertension via suppressing Hippo-YAP/PI3K/AKT signaling pathway. Front. Pharmacol. 12, 663551. 10.3389/fphar.2021.663551 33935785 PMC8082250

